# Know your enemy or find your friend?—Induction of IgA at mucosal surfaces

**DOI:** 10.1111/imr.13014

**Published:** 2021-07-30

**Authors:** Mats Bemark, Davide Angeletti

**Affiliations:** 1 Department of Microbiology and Immunology, Institute of Biomedicine, Sahlgrenska Academy, University of Gothenburg, Gothenburg, Sweden; 2 Department of Clinical Immunology and Transfusion Medicine, Region Västra Götaland, Sahlgrenska University Hospital, Gothenburg, Sweden

**Keywords:** commensal microbiota, IgA, infection, mucosa

## Abstract

Most antibodies produced in the body are of the IgA class. The dominant cell population producing them are plasma cells within the lamina propria of the gastrointestinal tract, but many IgA-producing cells are also found in the airways, within mammary tissues, the urogenital tract and inside the bone marrow. Most IgA antibodies are transported into the lumen by epithelial cells as part of the mucosal secretions, but they are also present in serum and other body fluids. A large part of the commensal microbiota in the gut is covered with IgA antibodies, and it has been demonstrated that this plays a role in maintaining a healthy balance between the host and the bacteria. However, IgA antibodies also play important roles in neutralizing pathogens in the gastrointestinal tract and the upper airways. The distinction between the two roles of IgA - protective and balance-maintaining - not only has implications on function but also on how the production is regulated. Here, we discuss these issues with a special focus on gut and airways.

## 1 Introduction

More IgA is produced than any other antibody class. Mucosal surfaces are filled with IgA-producing plasma cells that in humans produce several grams of antibodies every day.^1^ Mucosally produced IgA is transported through the epithelial cell layer into the lumen.^2^ Most of the production is secreted into the gastrointestinal tract and significant amounts are also found in nasal and lung fluids, saliva, tears, and breast milk.^3^ Secretion of antibodies at mucosal surfaces is an evolutionary preserved mechanism that hinders pathogens from invading tissues.^4,5^ As pathogens may encounter IgA before they reach any tissues, IgA can potentially give rise to sterile immunity by hindering the initial infection.^6^ Several studies of disease-causing viruses, bacteria, toxins, or adjuvanted antigens applied at mucosal surfaces have demonstrated that antigen-specific IgA responses are triggered against them.^7–10^ Airway viruses can also trigger antigen-specific IgA responses, an example being the ongoing SARS-CoV-2 pandemic; in diseased individuals antigen-specific IgA antibodies are often detected in serum before either IgM or IgG.^11,12^ At the same time, IgA is often described as a non-inflammatory antibody class that hinders inflammation, and it has been suggested that a significant proportion of IgA is produced in a similar fashion as natural IgM in the absence of typical antigen activation.^13^ Why so much IgA is produced, where and how the IgA response is initiated, and what are its triggers for it to be initiated has been the focus for research for many years but there are still many unknowns, and no consensus view has been universally accepted.

There are more IgA-producing plasma cells at mucosal sites than there are other plasma cells in the body.^1^ At mucosal surfaces, a joining (J) chain is added to IgA when it is synthesized, creating multimers, primarily dimers.^14,15^ After being secreted from the plasma cell, the dimers bind to the poly-Ig receptor (pIgR) that is expressed at the basolateral side of mucosal epithelial cells before being transported to the apical side of the cells where it is released into the lumen by proteolytic cleavage.^16^ This leaves a part of the pIgR receptor, called the secretory component, binding to the molecule which protects the complex from proteolytic degradation.^17^ Significant numbers of IgA-producing plasma cells are also found at non-mucosal sites, and IgA antibodies are present in the circulation.^18,19^ Although steady-state serum levels of IgG are four to five times higher than for IgA in healthy adults, the amounts produced are actually quite similar as the half-life of the classes differs three to four times.^20^ In addition, IgA antibodies are present in other body fluids such as within the cerebrospinal fluid.^21^ Unlike IgA produced at mucosal surfaces, serum IgA is predominantly monomeric and not joined to the J chain or the secretory component.^22^ Adding to the complexity, humans, but not mice, possess two IgA isotypes, IgA1 and IgA2, transcribed from two distinct heavy chain constant regions, with the first one dominating in serum and most tissues and the second more often secreted in the lower gastrointestinal along with IgA1.^23^ The two classes are similar to each other, but may have different stability and effector functions due to differences in the hinge region and glycosylation patterns.^24,25^ In similar with the gastrointestinal tract, the airways contain epithelial cells expressing pIgR that transport IgA into the lumen.^26,27^ This mechanism is likely more important in the upper airways as pIgR expression is evident in mucous, serous and ciliated epithelial cells in the lung but is virtually absent in alveolar cells.^28^ In fact, transudation of IgG may play a more important role than IgA secretion in lung.^29^

In addition to the role of IgA at the mucosa, other locations where IgA may play a role in health and disease have been suggested. A relatively large proportion of plasma cells in the bone marrow are IgA producing, both in mice and humans.^30,31^ These produce antibodies reactive against intestinal antigens and appear to form in the gut as a consequence of recognizing commensal bacteria or pathogens.^30,32,33^ This would allow these antibodies to protect the host against sepsis if the mucosal barrier breaks down.^30^ More recently, IgA-producing plasma cells were described within brain tissue, in particular along meningeal venous sinuses.^34–36^ These have been suggested to play roles both in autoimmune diseases, where they then may hinder overt inflammation by production of IL10, but also in protecting the host against infection into brain tissues. Clonal links have been found between IgA produced in gut and other tissues, and IgA produced at non-mucosal sites has been found to be reactive toward microbiota present in the gut. Finally, plasma cells are often present in tumor tissues, and in this case IgA expression have been associated with either worse or, at least in the case of ovarian cancer, better outcomes.^37,38^

Most mucosal surfaces maintain a commensal microbiota.^39^ This is particularly true for the gut, which in humans has been estimated to contain a total of 10^13^-10^14^ bacteria belonging to around 1000 different species.^40^ In addition, the gut supports fungi and viruses at steady state that also influence the mucosal environment.^41,42^ Similarly, both the upper and lower airways maintain specific microbiotas.^43^ Many diseases are associated with changes in the composition of the microbiota, and in some cases, such alterations may contribute to the development of disease.^44,45^ This constant presence of stimuli plays an important role in the maturation of the epithelium as well as the mucosal immune system.^46,47^ At the same time, the constant presence of bacteria presents the immune system with a difficult challenge—how to differentiate non—self-molecular structures derived from beneficial microbiota (that should be tolerated) from those coming from pathogens (that should trigger immune responses against the infection).

Recent studies have found that a large part of the microbiota in the gut is covered with IgA antibodies, both in mice and humans.^48–51^ Two different mechanisms have been proposed. According to one model, bacteria are covered with IgA to protect the host from them.^52^ In other words, when bacteria with the ability to cause inflammation, colonize the gut, these will be detected by the immune system and a specific IgA response triggered. Subsequently, the bacteria are covered with IgA to avoid invasion and inflammatory responses, which enable the host to retain these beneficial, but pro-inflammatory, commensal strains. According to the other model, the IgA cover will hinder strong inflammatory antigen-specific immune response against bacteria, and thereby ensures that a regulatory environment forms that is associated with the formation of regulatory T cells (Treg).^53^ The two models are not mutually exclusive. Nevertheless, they would suggest different pathways for the generation of IgA. In the first case, inflammatory bacterial strains trigger specific protective immune responses that will subsequently maintain a healthy balance, while in the second an ongoing, presumably rather unspecific, IgA production is needed to maintain a non-inflammatory state that ensure that inflammatory responses are not triggered. Although it is possible that this represents two different pathways for IgA induction, for example, T independent or dependent induction, the proposed pathways nevertheless demonstrate that the functions associated with IgA production are closely linked to how it is induced—whether most IgA is derived from highly specific immune responses that are similar to antigen-specific IgG responses or if it is constantly produced in the absence of any specific trigger in similar with natural IgM.

## 2 Inductive and Effector Sites for Mucosal IgA Responses

While IgG is characteristic for specific systemic humoral responses, IgA is the archetypal mucosal antibody class. It is generally accepted that the production of these antibody classes represents the outcomes from separate systemic and mucosal immune systems that differ in inductive and effector sites.^54,55^ Although often described as entirely separate, there is some overlap between them, and antigen-specific IgG is often encountered in serum after oral immunization.^7^ Inductive sites for systemic responses are found in the spleen and in the lymph system, where antigen and antigen-loaded dendritic cells arrive via blood or afferent lymph for B and T cells to react to antigens.^56^ Proteinaceous antigens trigger both B and CD4+ T cells and following interactions between these, germinal centers (GC) form within the lymphoid follicles as a consequence of expansion of rapidly proliferating antigen-specific B cells.^57,58^ The production of antigen-specific serum antibodies is subsequently achived when the activated B cells differentiate into short-lived local plasma cells or migrate to bone marrow to become more long-lived.^59–61^

**Figure 1:**
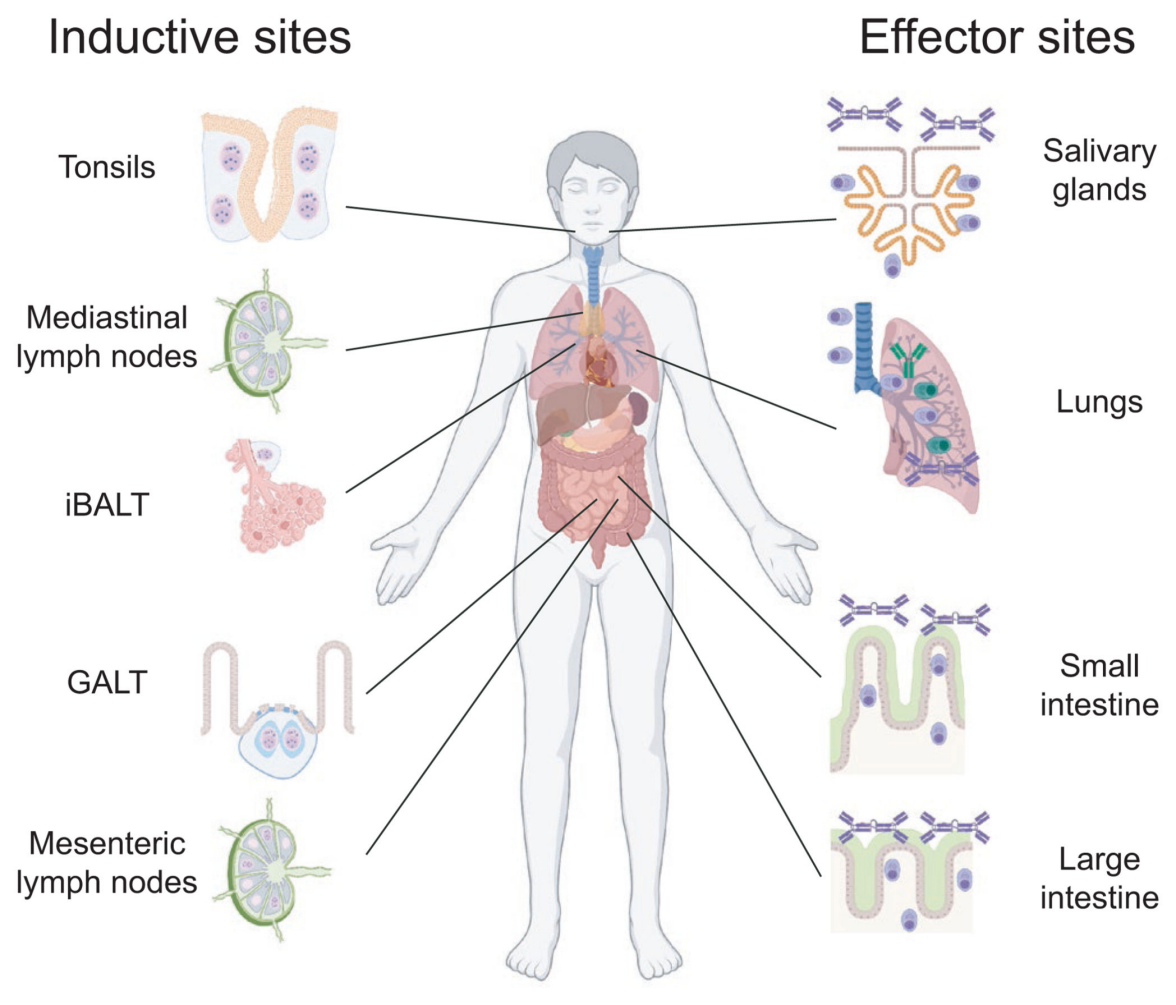
Inductive and effector sites for gut and airway antibody mucosal responses. The mucosal systems for immune responses in the airways and the intestinal tract contain several sites involved in activation of adaptive immune response (inductive sites) and production of antibodies (effector sites). Inductive sites for airways include tonsils (considered the nasal-associated lymphoid tissue (NALT) equivalent in humans) in the upper airways, inducible bronchus-associated lymphoid tissues (iBALT) in the lower airways and mediastinal lymph nodes (medLN) that drain the lungs. Gut-associated lymphoid tissues (GALT), primarily made up of Peyer’s patches (PP) and isolated lymphoid follicles (ILF), function as inductive sites in the gut while mesenteric lymph nodes (MLN) drain the gut. Plasma cells producing IgA antibodies are found both in the upper airways, exemplified here by a salivary gland, and lower airways where both IgA and IgG are produced, and represent airway effector sites. In the gut, the lamina propria of the large and small intestine function as an effector site and is filled with plasma cells that produce IgA antibodies

Inductive sites for the mucosal immune system, known as mucosal-associated lymphoid tissues (MALT), are non-encapsulated lymphoid follicles lacking afferent lymphatics that are embedded in the mucosa and submucosa (Figure 1).^54^ They can be further divided based on which areas they are found in, giving rise to inductive systems such as gut-associated lymphoid tissues (GALT), nasal-associated lymphoid tissues (NALT), or induced bronchial associated lymphoid tissues (iBALT).^62^ Distinct organ types make up each local system. Thus, GALT is made up of larger structures containing several lymphoid follicles (Peyer´s patches (PP) and colonic patches(CP)) and single follicles (isolated lymphoid follicles (ILF)), while NALT consist of several distinct tonsils (in humans) or structures along the nasopharyngeal wall (in mice).^63,64^ These structures are dependent on specific *anlage* formed during fetal life to develop, but differ between tissues and species with regard to whether they are dependent on microbiota to fully develop into mature tissues.^63–65^ In contrast, iBALT only develop as a consequence of inflammation and should possibly be considered a tertiary lymphoid organ.^66–68^ During inflammatory conditions, tertiary lymphoid tissues can also develop in the gut mucosa and these are hard to distinguish from ILF.^69–71^ To which extent tertiary tissues developing within the mucosa are distinct from those that form at non-mucosal inflammatory sites is not known.^72,73^ In addition to classical MALT, efferent lymph from the mucosa and MALT drain into lymph nodes, with, for example, mesenteric lymph nodes (MLN) receiving lymph from the gut and mediastinal lymph nodes (medLN) from the lungs.^74,75^ It is now appreciated that these lymph nodes should not be considered as one continuous organ; the MLN, for example, consist of several separate lymphoid follicles that handles antigens from different segments of the gut, and the arrival of an antigen to different follicles will influence whether it will trigger tolerogenic or inflammatory responses.^76^ Since lymph nodes are not situated within the mucosa and receive afferent lymph, they are not strictly part of the MALT but they still influence the induction of mucosal responses.

Unlike systemic lymphoid tissues, some MALT maintain GC in their B cell follicles in the absence of any immunization. This is, for example, the case for PP in mice and tonsils in humans.^63,77^ GC are also commonly present in MLN during steady-state conditions.^63,78^ In some animal species, for example, birds, rabbits, and sheep, GALT play a role in the generation of primary diversity of B cell receptors as random mutations or parts of upstream pseudogenes are introduced into the V regions, but in human and mice they are mainly involved in shaping specific immune responses.^79^ However, even in mice and humans, the gut has been suggested to take part in early B cell development, and both VDJ recombination and selection of transitional B cells into specific B cell lineages have been described.^80–83^ Unexpectedly, some recent single-cell RNASeq studies have also found gene expression patterns suggestive for early B cell development in the meningeal tissues of the CNS,^84,85^ with two studies even reporting a brain associated lymphopoietic niche.^86,87^ This observation raises the possibility that not only bone marrow, but also other tissues able to maintain long-lived plasma cells are able to support primary B lymphopoiesis.

Although the formation of GC in GALT is influenced by antigen-specific interactions with the microbiota, it does not seem to be the only force promoting their development. Germ-free (GF) mice develop GC in PP although they are smaller than in individuals with a microbiota.^69,78,88,89,90^ The same is true for mice expressing just a single rearranged transgenic B cell receptor or even lacking B cell receptors altogether, suggesting that neither antigen-specific signals nor B cell receptor-mediated uptake of antigen and MHC II presentation are always required.^91,92^ These B cell receptor-independent models still require T cell signals for GC formation in PP, which is also true in normal mice.^91–94^ Thus, while T cells and certain signals derived from them are needed for PP GC formation, archetypical cognate B-T interactions through the MHC II and T cell receptors appear to be dispensable. Regardless, there is a strong tendency to form GC in MALT, even in the absence of signals required in peripheral organs´ GC formation.

MALT are in general covered with a specialized follicle-associated epithelium (FAE) that allows for antigens to enter into the lymphoid structures below.^95–97^ This epithelium contains microfold (M) cells that, unlike other epithelial cells, can transport intact antigens from the mucosal lumen into the underlying tissues. Thus, instead of antigen entering through the efferent lymph as in lymph nodes, MALT will directly sample antigen from the lumen. This process is to some extent regulated, with molecular structures such as bacterial fimbriae, heat shock proteins, and IgA complexes promoting uptake, but other foreign antigens can also enter through unspecific processes.^2,98,99^ After entering, the antigens are not passively diffusing within the tissues. In GALT, for example, antigens enter into a specifically organized area named the subepithelial dome (SED) where DC, T, and B cells can directly interact with the M cells as well as with each other which ensures an optimal response.^2,95,98,99,100,101^ Given the rather non-specific function of M cells, non-proteinaceous macromolecules that contain microbe-associated molecular patterns (MAMP) will also enter. Thus, the MALT environment is distinct from that in lymph nodes or spleen; while these are thought to be essentially sterile and largely free from MAMP during steady-state conditions, MAMP are most likely ubiquitously present in MALT. Thus, it is unlikely that MAMP signals will function in a similar way as in systemic organs to determine whether there is an ongoing microbial invasion.^102^ In fact, even living bacteria may enter through the M cells into GALT, as has been shown for the commensal strain *Alcaligenes,* that colonize the PP after entering, and pathogens such as *Brucella Abortus* or *Salmonella,* that use this mechanism to get access to tissues.^103,104^

Major mucosal effector sites include the lamina propria along the gastrointestinal tract; lacrimal, nasal, and salivary glands in the upper airways; and mammary glands producing milk.^105^ In addition, cells producing secreted IgA are present within the lower airways and in the urogenital tract.^106^ Most IgA-producing mucosal plasma cells are thought to arrive to these effector tissues as plasmablasts that have left MALT after replicative expansion and class-switch recombination.^107^ The plasmablasts travel via the lymph system before they enter the bloodstream via ductus thoracicus and finally home to their effector sites. During steady state, IgA-producing plasmablasts dominate in blood compared to those expressing other classes, suggesting that they leave mucosal tissues continuously even when not reacting to novel pathogens or food antigen.^31^ The homing of these plasmablasts is controlled through expression integrins and chemokine receptors.^59^ In particular, the expression of integrin α4β1 and CCR10 has been associated with homing to mucosal surface with α4β7 and CCR10 specifically connected with gut responses.^108–110^

## 3 IgA Class-Switch Recombination

As for all antibody classes except IgM and IgD, class-switch recombination (CSR) is a prerequisite for IgA expression.^63^ This exchange of the heavy chain constant region from IgM to IgA occurs through a genomic deletion event that will still maintain the V region unaltered.^111^ Thus, antigen specificity is maintained while the functional characteristics of the antibody change. The process is controlled through regulated expression of activation-induced cytosine deaminase (AID), an enzyme that targets switch regions upstream of antibody heavy chain constant gene regions through deamination of cytosine into uracil within the DNA.^112^ Subsequently, the repair machinery that normally removes uracil residues introduced into DNA due to endogenous DNA damage will eliminate the AID-induced uracil; during this process, double-stranded breaks will develop in the switch regions that are specifically targeted by AID.^113–115^ An IgA expressing antibody gene is created when a break in the switch region upstream of IgM is joined to one occurring in the switch region upstream of IgA and the intervening part is deleted. This deletion process relies on the non-homologous end-joining machinery, likely as a consequence of cohesin meditated positioning of the switch regions with double-stranded breaks.^116^

The AID enzyme is also responsible for targeting V regions for mutations during GC proliferation, although in this case, the repair pathways used are slightly different than during CSR, resulting in point mutations rather than double-stranded DNA breaks. Importantly, both for CSR and mutation induction, DNA replication is required.^112,117^ Thus, it is unlikely that non-proliferating B cells will be able to undergo class-switch recombination or introduce mutations into their V regions.^118,119^

For CSR to occur, functional AID must be present in the cell nucleus, an even that is tightly regulated through several mechanisms to ensure that it only happens in proliferating B cells.^120^ The most critical signal for induction and activation of AID is transmitted through the surface receptor CD40, although other signals, such as MAMP recognition by TLR or BAFF receptor family members may substitute in some cases.^120,121^ These signals all activate NF-κB pathways that are likely critical in the activation process.^122^ Local cytokines act as cues that command CSR into different isotypes by activating sterile germline transcripts that open up the switch regions upstream of the antibody constant genes and make them targets for AID and position the constant regions.^116,117^ TGF-β has been closely linked to both induction of sterile IgA transcripts and for cells to undergo CSR to IgA,^123^ but signals from the two related cytokines a proliferation-inducing ligand (APRIL) or B-cell activating factor (BAFF) that activate the transmembrane activator and calcium modulator and cyclophilin ligand interactor (TACI) may also be able to promote the process.^124,125^ Other signals in the local milieu, such as interleukin 21 (IL21) and all-trans-retinoic acid (RA) play additional roles.^126–128^

As PP are surrounded by IgA-producing plasma cells in the mucosa, it has been assumed that CSR in GALT is efficiently skewed toward IgA. At the same time, IgA CSR appear to be rare in other organs. Recent observations have challenged the conclusion that IgA is the only or even major antibody class produced in MALT. In tonsils, for example, many IgG switched cells are present, although the mucosa in the upper airways, in similar with the gut, are dominated by IgA-producing plasma cells.^77^ In addition, in human GALT, as many as 10% of all B cells express IgG, and an evaluation of antibody heavy chain gene transcripts revealed that IgG transcripts were present in both sorted GC and memory B cells.^77^ Similarly, in mice relatively high numbers of IgG switched cells were detected in PP using single-cell RNASeq (Komban et al, manuscript in preparation). Recent studies have started to reveal signals needed for IgA CSR to occur in MALT. For example, in PP from mice with a normal microbiota, IgA dominated with some levels of IgG2b also being produced, whereas in germ-free animals or in animals with a limited bacterial microbiota, IgG1 was the dominant antibody class in PP with few IgA-switched cells detected.^78^ Skewing was also observed in experiments using microbiota transfers, where germ-free mice receiving microbiota from T cell-deficient mice reconstituted with Treg cells showed efficient induction of IgA, while in germ-free mice reconstituted with microbiota from T cell-deficient mice into which naïve CD4 cells had been transferred promoted IgG1.^51^ These observations are also in line with observations we made in transfer experiments with sorted T cells into nude mice. In this case, strong IgG responses were triggered in GALT after oral immunization in mice transferred with CD25-, presumably non-regulatory, T cells while if CD25+ T regulatory cells were co-transferred, the recipient mice could be immunized to produce IgA.^129^ In this model, the ability of T regulatory cells to produce active TGF-β was the factor needed to induce IgA production. However, other factors may also be involved, as both retinoic acid and IL21 have been shown to inhibit IgG switching when the cells are induced to IgA switching with TGF-β *in vitro*.^130,131^ Further investigations of the role for switch factors and cell types for efficient induction of IgA switching in MALT are clearly called for, as it seems that switching to other classes represents the default pathway in many cases.

## 4 T Dependent and Independent IgA Responses

An important division of systemic B responses is whether they are triggered by thymus (or T cell) dependent and independent antigens.^132^ Originally, this division was based on the observation that certain purified antigens, that is, proteins, required the thymus to give an optimal response, while others, that is, lipopolysaccharides (LPS) or highly repetitive antigens, did not.^133,134^ Eventually, the original observations led to the current view that it is whether the B cell interact with CD4 T cells through CD40-CD40L signals or not that determined the type of response—resulting in the term T cell-dependent or independent responses.^135^ Several studies have reported that B cells belonging to B1 and B2 marginal zone lineages are more often involved in T independent responses and B cells belonging to the follicular B2 lineage are more often involved in T dependent reactions.^136^ The classical view is that there are large differences in response triggered by the two types of antigens; in the case of a T dependent proteinaceous antigen, the B cell will proliferate in GC, will undergo somatic hypermutation of V regions and selection leading to affinity maturation of surviving cells, undergo class-switch recombination to other classes than IgM and will generate long-lived plasma and memory B cells, while in the case of a T independent antigen there will be proliferation of B cells but no GC formation, production of IgM of low affinity and that most plasma cells will be short-lived.^132^ Therefore, class switching, mutations of V regions, memory cell development, and long-lived antibody responses are all considered sign of T cell-dependent reactions, while a lack of these signs is associated with a T independent process. However, it should be pointed out that the division is a bit artificial in most infectious models, as essentially all pathogens will contain a mixture of T cell-dependent and independent antigens, often even covalently linked to each other, giving rise to a mixed responses where some T independent epitopes may give rise to T dependent responses. Nevertheless, one area where the distinction between the two types is important even clinically is during the development of subunit vaccines; in these polysaccharides are sometimes conjugated to proteins is to give more long-lived T dependent responses.^137,138^

Whereas the differences between T dependent and non-dependent responses to systemic antigens have been extensively characterized, it is much more problematic to define whether they should be seen as two separate arms in the mucosa.^63^ As IgA is a class-switched antibody, it must be assumed that cells expressing IgA have proliferated while they expressed AID at some time during their development. Hence, the most straight-forward assumption would be that all IgA-producing plasma cells in gut, expressing a switched antibody and being highly mutated, would have passed through GC in GALT. However, several mouse models and observations in humans have been made that in fact support the view that IgA production can be initiated in T independent systems (Table 1). Still, essentially all such observations come with caveats that suggest that T dependent reactions may non-the-less dominate in most cases.

Taken together, from these studies it can be concluded that in individuals lacking certain immunological pathways, IgA production in the gut can be T cell independent but that T dependent induction will totally dominate in immunoproficient individuals. Nevertheless, as will be discussed later, it is possible to imagine the existence of responses that are in between what is normally defined as T dependent and independent responses in MALT. The fact that high levels of mutations are found in antibody V regions from IgA secreting gut plasma cells in humans and mice demonstrate that the cells must have proliferated.^139–141^ Thus, the lack of data suggesting that extensive B cell proliferation occurs outside of organized lymphoid tissues make it unlikely that the vast amount of IgA plasmablasts continuously produced can be generated elsewhere.^142^ It should be noted that many early studies supporting that the lamina propria was an important site for IgA generation were performed before ILF had been defined as an entity separate from PP, and more recent studies have failed to identify molecular markers suggestive of ongoing CSR outside of organized lymphoid tissues such as ILF.^140,142,143,144^ Nevertheless, it is hard to exclude that T independent IgA CSR never occurs outside of GALT, although it must be concluded that in that case, it will only make a minor contribution to gut secreted IgA.

## 5 Antigen-Specific IgA Induction in Peyer’s Patches using NP-CT as a Model Antigen

We have developed a hapten-carrier system that allows us to track T cell-dependent antigen-specific B cell responses in PP following oral immunization.^145^ A traceable antigen was created by conjugating the well-known hapten 4-hydroxy-3-nitrophenyl (NP) to cholera toxin (CT) that act as a carrier to create an NP-CT conjugate. After oral immunization, CT triggers strong T cell-dependent responses in the gut,^146^ and we found that this was also the case for NP-CT.^145^ As possibly the most used hapten, NP has been extensively used to study antigen-specific antibody responses after systemic immunization, in particular, in C57 BL/6 mice.^147^ We adapted some of the well-established assays that exist for examining NP responses to be able to study gut responses. In a first study using NP-CT as an antigen, we were able to demonstrate that the response against a single antigen could be highly efficient, with 15% of all IgA plasma cells being reactive against NP and 30% against CT after repeated oral immunizations. Furthermore, we found that antigen-specific B cells invaded already existing GC in PP after the immunization, that affinity maturation was highly efficient, and that there was an exchange of cells not only between PP and effector sites but also between different inductive sites.^145,148^ Together these observations suggested that responses were coordinated by exchange of cells between tissues and that this helped to synchronize the response ensured that high-affinity clones generated in one PP could spread and "take over" GC in other PP lacking high-affinity clones.^75^

We had previously observed that CSR to IgA can occur before B cells enter GC in PP, and Reboldi *et al*^149^ from the laboratory of Jason Cyster later made the same observation.^140,150^ At first, this seemed to indicate that CSR to IgA in PP differed from that to IgG in systemic organs as the latter was thought to mainly occur in the GC, but it was in fact later demonstrated that CSR to IgG also often happens before B cells form GC and undergo somatic hypermutation.^151^ Reboldi *et al* also demonstrated that the area where pre-GC IgA CSR occurred was the SED region, where antigens enter PP through M cell-mediated transport and suggested that B cells will first migrate to the SED upon entering the PP and after that into GC after CSR. This observation, and that we found a subpopulation of activated B cells expressing CCR6, led us to study the SED region in some more detail using our antigen-specific system.^101^ Employing NP-CT as an oral antigen and transfer of Vh1-8^hi^ expressing NP-reactive B cells that were labeled with GFP, we found that antigen-specific cells were not only present in SED at the start of the response, before cells entered the GC. Instead, all through the response, the GC and the SED regions harbored proliferating antigen-specific B cells.^101,152^ While B cell proliferation in SED was less dependent on antigen affinity than the subsequent entry into GC and proliferation within it, the presence of T cells supported SED proliferation.^152^ Nevertheless, some level of proliferation was maintained even when T cells were depleted, suggesting that CSR may be ongoing even in the absence of T cells. This was in line with the previous report from Reboldi *et al* that suggested that dendritic cells may play an important role in this process.^150^ Such proliferation may be important during the early response to antigen, since very few antigen-specific T cells have been activated at this time, while T cells are more important later during the response. However, pre-GC B cell proliferation in the SED region of the PP may be sufficient for IgA CSR even in the absence of T cells but will likely result in plasma cells with very limited numbers of mutations in their V regions because of limited replication and absence of specific T cell cues. Although it has not been directly tested yet, such a T independent pathway could be the one leading to IgA production in humans and mice lacking CD40 signals, which would explain the lack of V region mutations in IgA cells from such individuals.^140,154,155^

In mice lacking the SAP-SLAM pathway, CD40 signals are present, but traditional cognate interactions between B and T cells cannot occur^154^; in the laboratory of Ziv Shulman, Biram *et al* found that in these knock-out mice, PP GC will form but that in the absence of efficient interactions between B and T cells, antigen-specific cells will not enter into the GC after oral immunization and that the selection of B cells within the PP is disturbed.^152,156^ The situation in these mice is distinct to that in CD40 deficient mice that totally lack GC.^93^ Thus, while CD40 signals are strictly required for GC formation in GALT, traditional cognate B-T cell interactions involving the SAP-SLAM pathways are necessary for efficient responses as they facilitate entry into GC and/or selection of high-affinity clones. Nevertheless, even in the absence of SAP-SLAM interactions, some B cells will manage to form GC in PP.

So why are there B cells in the SED region even when cells belonging to the same clone have already invaded the GC and started to mature there? Some of our observations give some clues. It is likely that antigen is a very limiting factor in most gut responses. A single antigen is diluted enormously in the sea of other antigens present in the gut, and the local level in PP is further limited by the M cell-mediated transport. Then, how can an antigen like CT give strong responses with affinity maturation, after an oral doses of only 10μg? As mentioned above, we found that activated antigen-specific B cells were present in the SED throughout the response, often in very close contact with M cells.^101^ However, they were not stationary there. By visualizing the tissue with 3D microscopy, we always observed some cells between the SED and GC, even several days after immunization.^152^ When we instead studied SED cells using live imaging, we also observed that the turnover of cells in the SED was relatively high and that cells in fact moved from the SED towards the GC.^101^ In addition, when migrating B cells had access to antigen, they would bind to this and transport it from the SED and toward the GC, possibly positioning it on their cell surface rather than degrading it. Careful sequencing of antibody genes from SED and GC cells also supported that such movements occurred.^152^ Thus, it appears that one important function of the cells in the SED is to bind to specific antigens to ensure that the GC is loaded with antigen.^75^ In fact, previous findings show that B cells in both LN and spleen can unspecifically transport antigen to GC using complement receptors, and transport of specific antigen recognized by the B cell receptor has been suggested to be important between lung and spleen.^157,158^ Thus, the SED region may act as a site that ensures that B cells transport antigens to FDC network of the GC when the antigen is present in the local gut environment, and attract newly arrived naive or activated B cells to enter the GC to compete for high-affinity binding.^75^

**Figure 2:**
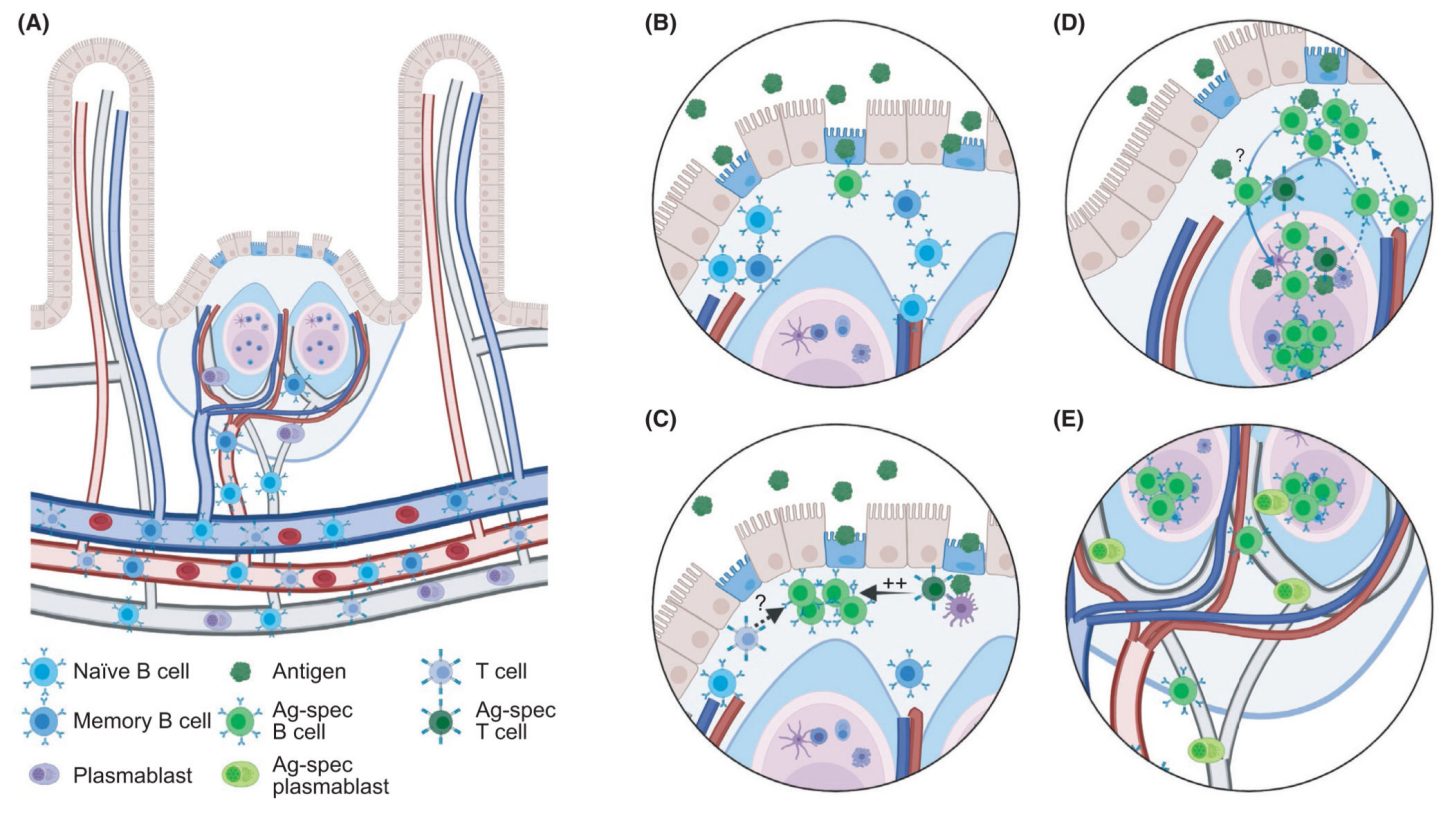
Antigen-specific B cell responses in Peyer´s patches. (A) Peyer´s patches (PP) are supported by blood and lymph vessels that enables B and T cells to enter via high endothelial venules in areas between B cell follicles.^237–239^ These, as well as plasmablasts generated during the response, leave the organ via the lymph that subsequently pass the mesenteric lymph nodes before returning to blood when the thoracic ducts enters into the left subclavian vein. (B) Naive and memory B cells will enter the PP and migrate toward the subepithelial dome (SED) where they encounter antigens transported into the structure through M cells. When an antigen-specific naive or memory B cell encounters antigen, it will be activated in a process that is not dependent on antigen interactions but do not require high affinity. (C) Following activation, antigen-specific B cell will proliferate within the SED. The process is expedited if they interact with antigen-specific T cells and it may also benefit from non-cognate interactions between B and T cells. (D) Some activated B cells will enter into the preformed germinal centers (GC) where they will continue to proliferate, but SED proliferation will still be maintained. Entry and further proliferation within the GC will be dependent on antigen affinity as well as cognate interactions with T cells. B cells migrating from the SED may carry specific antigens using their B cell receptors that are loaded onto follicular dendritic cells in the GC. B cells showing signs of already having been in the GC are also present in the SED. It is not known if these migrate directly from the GC or leave as activated memory cells that subsequently reenter to the SED from blood. (E) Antigen-specific B cells and plasmablasts can leave the PP through the lymph. During the response, many of the B cells leaving appear to still have an activated phenotype

Based on these studies, a rather complex chain of the events during T dependent responses to oral antigens appear to take place (Figure 2). When entering the PP from blood, naive B cells will first enter the SED region, most likely in the absence of cognate T cell help. Here, they are tested for their ability to interact with antigens entering the SED through the M cells; only cells that bind antigen will start to proliferate and undergo IgA CSR. In the absence of cognate T cell help or if the cells are of low affinity, the SED response will wane over time, resulting in IgA-switched cells with low number of mutations in their V regions that subsequently become plasmablasts that can leave GALT to migrate to the mucosa. On the other hand, if cells have sufficient affinities and interact efficiently with T cells that recognize the same antigens, they will migrate toward the GC, where the cells will continue to proliferate and undergo affinity maturation. Some cells responding in the absence of cognate T cell interactions may still make it into the GC where additional proliferation will occur, a process that may still require non-cognate interactions with T cells (see below). In addition, some activated B cells will leave the GC, either to return to the SED, where they will reencounter antigens that can be transported back to the GC or to leave via the lymph, which will allow them to enter other PP where they are tested in the SED for their ability to interact with other local antigens. This process will allow for synchronization between spatially distinct GALT structures.

## 6 TI Antigens and GC Responses

When cognate B-T cell interactions occur during T dependent responses in PP, B cell proliferation increases in SED, and B cells with sufficiently high affinity are able to enter GC and give rise to highly mutated plasma and memory cells.^152^ Then, what about T independent antigens, how do they trigger PP responses? And why do IgA antibodies specific for them carry mutations in their V regions^90,159,160^? One possibility is that essentially all microbes are detected by the immune system as intact immunogenic particles that contain both antigens normally considered T independent and dependent. Hence, even when these antigens are not covalently linked, B cell receptors on the cell surface will trigger antigen uptake of the whole particle and present peptides derived from any protein within the microbe. Another possibility is that B cells activated against T independent antigens can actually enter GC in PP the absence of cognate B-T cell interactions.^63,161^ GALT is rather unique compared to other lymphoid organs; during systemic responses GC form following cognate T-B interactions but in GALT GC are already established when an antigen is encountered. Could it be that the cues that B cells need to enter an existing GC are different from those needed to initiate the formation of a GC? Several observations from systemic organs appear to support this hypothesis. For example, it was observed that activated B cells started to form rudimentary GC in the absence of cognate B-T interactions but that these collapsed if T cell help was not present.^162^ Possibly, if T cells reactive against other antigens maintain the GC, these newly activated B cells may survive longer. Furthermore, it has been demonstrated that B cells recognizing other antigens than those which triggered GC formation can enter GC after activation. While pre-existing T cells, able to provide cognate help to B cells, can facilitate this process, they are not indispensable for cells to enter.^163,164^ It is also supported also by the phenomenon of epitope spreading during autoimmune responses. Here, a response is started against a single epitope but is then spreading to non-related epitopes, as it has been demonstrated in an SLE mouse model.^165^ In this model, a knock-in of both a heavy and a light chain reactive against ribonuclear complexes made a proportion of the B cells auto-reactive, which resulted in spontaneous GC formation. However, the autoimmune response gradually spread to unrelated epitopes, presumably through invasion of these GC by B cells having other antigen specificities. Finally, non—antigen-specific non-activated B cells appear to be able to access the GC environment, possibly in order to evaluate their ability to recognize antigens within it, and recent experiments suggest that such bystander cells may sometimes even undergo diversification in GC.^163,166^ Thus, although not proven, it would seem feasible that T independent antigens can trigger GC entry of B cells in the PP and at least some proliferation within it, which could explain how mutations are introduced into their V regions.

The processes of somatic hypermutation and selecting high-affinity clones occur spatiotemporally distinct in the GC, occurring in the dark and light zones, respectively.^58,167^ However, even though typical T dependent antigens will give rise to efficient affinity maturation in PP, this process may be less efficient or even absent when T independent antigens are involved.^145,161^ One study that supported this idea found that antibodies that bound to the surface of microbiota, which to a large extent are made up of polysaccharides, did not show any notable decrease in binding when all mutations were removed from their V regions.^168^ Thus, in this study, germ-line antibodies, presumably present in naïve B cells, against T independent antigens have similar affinities as those produced in mutated post-GC B cells. However, other recent studies have instead found that mutations in IgA antibody genes do promote binding even to antigens classically defined as T independent. In these, binding of cloned antibodies to specific glycol-epitopes have been studied in humans and mice, and the conclusion was that high-affinity binding to specific epitopes did require the mutations to be present.^90,160^ Furthermore, mutation-dependent binding to the surface of microbiota, an area that is often heavily glycosylated, has been described both when antibodies from GALT B cells or LP plasma cells were cloned.^78,169,170^ Thus, although the role of affinity maturation to T independent antigens in the gut is still debated, most recent studies suggest that these antigens do induce affinity maturation when present on commensal microbiota.

## 7 T Cells Contributing to IgA Responses

As discussed above, the role of T dependent and independent antigens in gut responses cannot easily be answered by just comparing responses in mice lacking T cells with that in normal mice as redundancy will skew the results. Furthermore, to fully appreciate the role that T cell signals play in allowing GC to form versus in the activation of individual B cells and their entry into pre-existing GC, experiments must be done in mice where GC already exist but where activated B cells cannot access T cell help. However, what is clear, is that, in adult individuals, most IgA-producing antibodies plasma cells carry mutations in their V regions and for this reason, they must have proliferated extensively, most likely in a GC.

So, which T cells need to be present in PP or other MALT to form GC? In systemic organs, induction of T cells that enter into the T follicular helper (Tfh) lineage characterized by Bcl6 expression is critical for GC formation.^171^ But is that also true in MALT? In fact, there have been reports suggesting that Bcl6 expression may not even be needed for antigen-specific gut responses to T dependent antigens.^172^ And even if Tfh cells are needed—will they develop from naive T cells, or could other T-helper lineages develop into Tfh cells in MALT? Different types of Tfh cells that share phenotype with other lineages have been described, making them Tfh1, Tfh2, or Tfh17 cells; do these develop when Tfh cells differentiated toward other phenotypes or do Th1, Th2, and Th17 cells become Tfh cells.^173,174^ And what about T follicular regulatory (Tfr) cells—they seem to develop primarily from thymic T regulatory cells but can they also be derived from induced regulatory T cells or even naive cells.^175^

Some studies have suggested that antigen-specific IgA responses in PP may involve substantial cross-differentiation. Initially, Treg cells were implicated. In an antigen-specific mouse model recognizing bacterial fimbriae, depletion of Treg cells decreased IgA production and this could subsequently be rescued by transfer of CD25+ or Fox3 expressing cells.^176^ Furthermore, transfer or purified FoxP3-expressing Treg cells from spleen and lymph nodes into CD3ε-deficient mice lacking T cells led to a relative rapid induction of IgA production as well as GC formation with T cells within the PP.^177^ These transferred cells appeared to downregulate FoxP3 expression before becoming Tfh cells, making the pathway hard to track in the WT situation since lineage tracer mice were not used. Notably, later experiments revealed that the FoxP3-expressing cells did not only differentiate into Tfh cells but also directly regulated IgA response as Tfr cells in the transfer system, suggesting that Tfh and Tfr could belong to the same clones, making the two functions somewhat hard to distinguish from each other.^51^

Other experiments instead suggested that Th17 cells could be involved. It was first suggested that IL17 was needed for efficient mucosal responses and linked this to induction of Th17 cells.^178^ However, subsequent data suggested that this may be due to that IL17 supported pIgR-mediated transport of IgA rather than induction of B cells, but also that IL21 production from Th17 cells may play a role.^127,179^ Finally, using a tracer model it was shown that cells that had previously expressed IL17 may cross-differentiate into Bcl6 expressing Tfh phenotype that supported IgA CSR after transfer into mice lacking T cells.^180^

So how can these apparently contradictory results, that both Treg and Th17 cells develop into Tfh cells in PP, be integrated into a model for IgA responses in the normal situation. Is there competition between theses pathways? Or are they both minor pathways showing what can happen in artificial systems rather than indicators of what do happen in non-modified individuals? And if Th17 cells cross-differentiation is required, what would happen when a novel antigen is encountered? Would cells go through a short Th17 stage during the primary response or would we only be able to respond to previously encounted antigens? After all, T cells only become Th17 cells after they encounter antigens.

We recently started to address some of these questions by studying the roles of different T cell subsets in the response to specific antigens. A first study casted some light on the role of Treg and Tfh cells.^129^ This study was based on the fortuitous observation that in transgenic mice carrying a T cell receptor reactive against chicken ovalbumin, adjuvants were not needed for induction of specific IgA and IgG responses after an oral immunization with ovalbumin. This was also the case when T cells from the transgenic mice were transferred into T cell-deficient animals, and while CD25-non-regulatory T cells were sufficient for IgG responses, both non-regulatory and CD25+ Treg cells were required for IgA responses. In this transfer model, for the non-regulatory cells to support immunization in the absence of adjuvant, they had to be isolated from mice with a microbiota, and we found that the presence of a microbiota led to the occurrence of T cells in PP that expressed a recombined endogenous T cell receptor in addition to the transgenic T cell receptor. Thus, it seemed that the endogenous receptor detected antigens from the microbiota and that the transgene at the same time allowed for the cells to support the response against ovalbumin. A large fraction of these transferred non-regulatory cells expressed the markers PD-1, CXCR5, and Bcl6, suggesting that they were *bona fide* Tfh cells, and they also maintained this phenotype after transfer. At the same time, transferred CD25+ T regulatory cells did not have to express dual T cell receptors, and it was sufficient to transfer splenic, non-transgenic thymus-derived T regulatory cells to support IgA induction. Their role appeared to be to provide TGFβ and possibly other factors to support IgA CSR. Overall, this specific model suggested that when the ovalbumin-specific Tfh cells were at a non-limiting level in PP, due to constant activation through antigen-detection of the microbiota through expression of endogenous T cell receptors, adjuvants were not required for oral immunization. Furthermore, in the model thymic derived T regulatory cells were required to provide signals critical for the IgA CSR but this did not require antigen interactions.

This model cast some light into the role of T cells but was limited in similar manners as previous studies by being based on non-natural circumstances that may skew the results. We have more recently been addressing the role of T cells play in GALT in a more physiological system that respond to a novel antigen in a more natural polyclonal situation (Gribonika et al, under revision). In this study, WT mice were immunized orally with CT, and an MHC II tetramer loaded with an immunodominant peptide from CT is used to identify antigen-specific cells that are subsequently investigated using multicolor flow cytometry and single-cell RNASeq sequencing coupled to cloning of T cell receptor genes.^181,182^ Using this approach, we did not find any support for the notion that antigen-specific Treg or Th17 cells were involved in CT-specific T cell responses or cross-differentiated to Tfh cells. Rather, cells with a Tfh phenotype dominated the antigen-specific response and did not share clonal relationships with Th17- or Treg-like cells, and a lineage tracer showed that the Tfh cells had not previously expressed IL17. Furthermore, we found that thymus-derived Treg populations shared transcriptional characteristic with Tfr cells, but had minimal clonal relationships and only minor transcriptional similarities with Tfh cells. The situation was very similar when steady-state non-selected T cells from PP were analyzed using single-cell RNASeq; Tfh-like cells made up more than 50% of all memory T cells and they showed very limited T cell receptor sharing with any other T cell lineages. Thus, although different transfer models have indicated that Th17 and Treg cells have the potential to cross-differentiate into the Tfh lineage in PP, we found little evidence for transcriptional or clonal relationships between the lineages in normal adult mice, neither for steady-state T cells nor cells responding to a novel antigen. Rather, the analysis suggested that direct differentiation of naïve CD4 T cells into the Tfh lineage totally dominated the response.

## 8 Do Model Antigen Responses in PP Reflect Responses to Pathogens and Commensal Microbiota?

With these observations using model antigens, an important question is how well they reflect responses to pathogens or other gut antigens. The responses that are triggered by CT and other related toxins represent a golden standard for mucosal vaccinology as they give strong protective responses made up of both secreted IgA and serum IgG, long-term production of antibodies, and development of long-lived memory B cells.^146^ However, although some mucosal vaccines are available, further development is needed to give responses of a similar magnitude, and these should ideally occur in the absence of the side effects associated with CT.^7^ Thus, in mice that tolerate CT better than humans, a CT-like response is a reasonable goal for mucosal vaccine response, both when it comes to levels of antibodies produced and types of cells induced. Similarly, certain mucosal infections give rise to long-term protection after infection, while others do not.^8,10^ To understand give long-term protection, it is likely that CT will represent a relatively good model.

**Figure 3:**
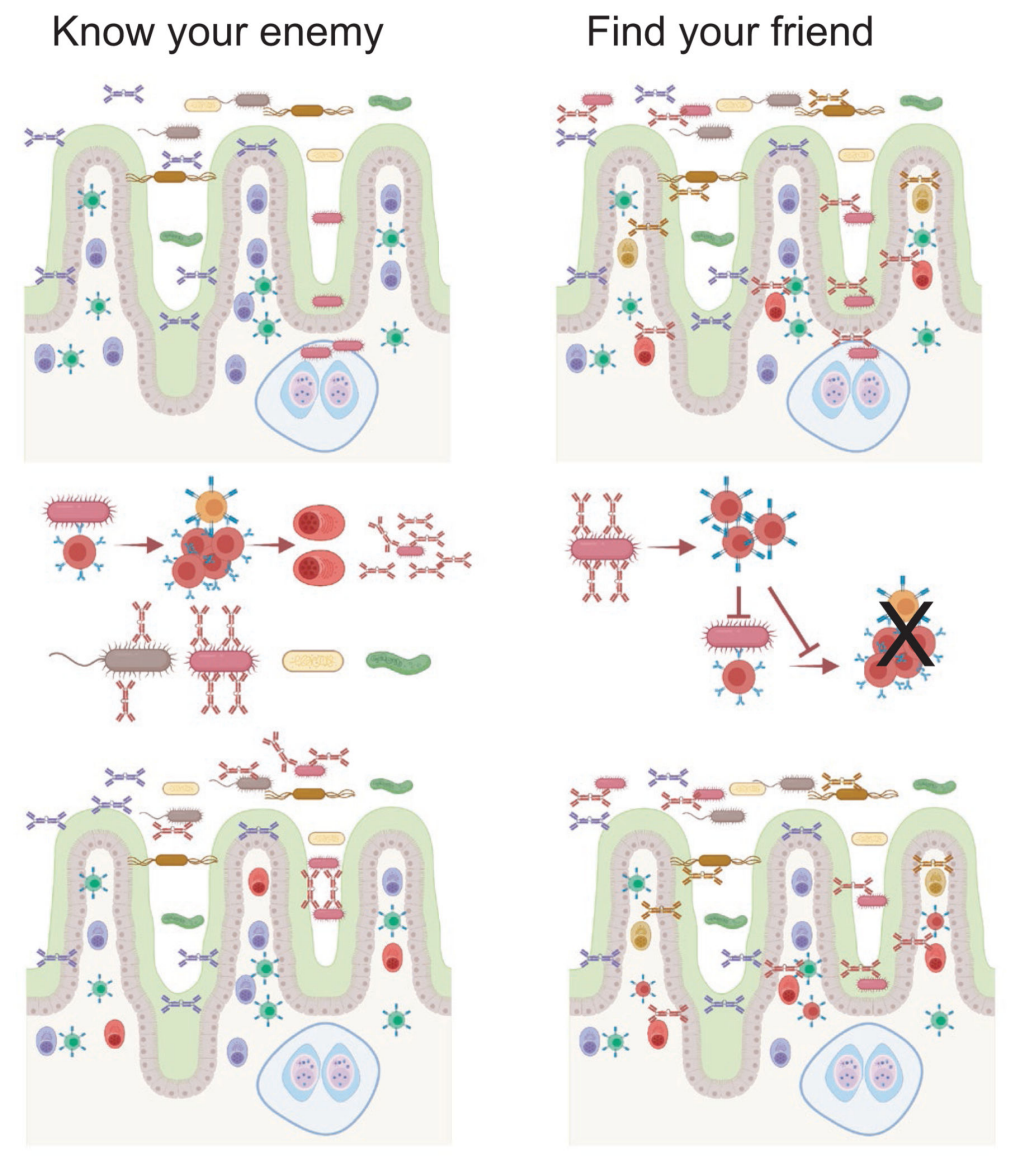
Commensal binding of IgA antibodies and consequences of the induction pathways. Two mutually non-exclusive models for IgA binding to commensal microbiota has been proposed, and these have implications on the induction of antibodies. According to a "Know your enemy" model, bacterial strains able to trigger inflammation will activate antigen-specific responses in GALT, likely through T cell-dependent mechanisms, which ensures that this bacterial strain cannot invade into tissues again. IgA antibodies will cover other bacterial strains due to cross-reactivity between epitopes. According to the "Find your friend" model, broadly reactive IgA antibodies are produced in a T independent manner before the bacterial encounter, possibly even in the absence of antigen-specific interactions due to triggering of pattern recognition receptors on B cells. Bacteria covered with non-inflammatory IgA will trigger regulatory T cell responses if the bacteria manage to invade into tissue, which in turn ensures that specific responses or inflammation are not triggered

When it comes to the IgA that covers the microbiota two schools of thought have developed (Figure 3). One favors the view that the IgA production represents a highly specific response to bacterial strains which may cause inflammation if they are not covered with IgA^52^; the other suggests that IgA binding to the microbiota is made in a rather unspecific manner in the absence of T cells help and thus hinders any inflammation, a role a bit akin to that of natural IgM.^48,53^ An argument for the latter view is that a large proportion of the gut bacterial microbiota is covered with IgA, and although some of these strains can cause inflammation, most will not. Another is that some cloned antibodies were of low affinity for the bacterial antigens.^48^ While these arguments are valid, it is important to consider the fact that antibacterial antibodies will mostly bind to polysaccharides on the bacterial surface.^183^ Although there is significant diversity among polysaccharides, many bacterial strains will carry identical or similar structures, and antibodies reactive to one strain will also be reactive against other strains.^78,160^ Furthermore, low-affinity binding to one structure cannot exclude high-affinity binding to another.^184^ Thus, the ability of the IgA antibodies to bind to many different bacterial strains may be a result of the fact that their production is induced by one specific strain, which can cause inflammation, but later antibodies from the same B cell clone will cross-react, possibly with lower affinity, with large number of other bacteria. When individual gut IgA antibodies were cloned from single mice or human donors, many of them were indeed able to bind to a relatively large proportion of gut microbiota representing many phyla.^168,169^ Thus, the pattern of binding is compatible with the hypothesis that a response against a specific structure will generate a cross-reactive response against similar structures on other bacterial strains. In line with this, some of these studies found that when Ig genes cloned from PP or plasmablasts were reversed to germ line by removing mutations binding was lost or diminished.^78,90,160,169^ This suggests affinity selection after introduction of mutations and would imply a role for GC in the process. One study did not see a correlation between mutations and binding, and also reported that both T cell-deficient and germ-free mice fed an antigen-free diet produced antibodies able to bind to bacterial antigens despite having never encountered them.^168^ How to interpret this was unclear until recently, when two studies have shed some light on the issue as they both found specific antibody clones enriched in PP GC of germ-free mice, possibly due to increased probability for recombination and reactivity against self-antigens.^78,90^ Thus, there was selection in GC of these clones even in germ-free mice, which can explain the presence of such clones in the gut. Taken together, current data would support a constant selection of B cell clones in PP GC. This will mostly be driven by antigens from the microbiota and will result in both affinity maturation but cross-reactivity to similar structures on other bacterial strains will still be maintained. The fact that essentially all IgA antibodies in gut are mutated certainly suggests that the cells producing them had passed through GC with PP being the most likely organ^139,140,141,185^; otherwise, a distinct, so far unidentified, area where IgA cells undergo rapid proliferation and acquisition of mutations must be posited.

Overall, the outcome from reacting to commensal microbial antigens thus appears to be similar as the one we have observed using the NP-CT model antigen. Nevertheless, this does not necessary mean that the process of activation and selection is identical. After all, CT is an incredibly powerful antigen that also functions as an adjuvant.^186^ Maybe the toxin can directly influence B or T cells so that these are reacting in a different manner than those activated against other antigens? Recent experiments using single-cell RNASeq analysis of activated lymphocytes present in normal PP or antigen-specific cells reacting to CT antigens after oral immunization have been conducted in our laboratory. As discussed above, T cells were identified by a MHC II tetramer carrying an immunodominant peptide from CT^181^ whereas the B cells used in the analysis were GFP-expressing B1-8^hi^ cells activated by NP-CT immunization (Gribonika et al, under revision; Komban et al, manuscript in preparation). The insights reached from these studies will be discussed in detail in upcoming publications, but what is clear from the studies is that the transcriptomic analysis does not identify extensive differences between B and T cells activated against CT antigens compared to other activated cells in the PP during steady state. Thus, although CT is without any doubt a powerful oral antigen, able to replace almost half of all IgA-producing plasma cells in the gut following repeated immunizations,^145^ there is no reason to believe that B or T cells would be activated through an non-physiological process in the response.

## 9 Mucosal Responses to Airway Infections

Much interest has focused on gut IgA production and responses during the last decades, both due to that the gut is the major IgA-producing organ and to an increased interest in the importance of the gut microbiota and immune responses against it. However, the still ongoing SARS-CoV-2 pandemic has put airborne pathogens and the antibody responses against them into the spotlight. The airways are arguably the most important entry sites for pandemic, epidemic, and endemic pathogens,^187^ and local secretion of airway antibodies has the potential to create truly sterilizing immunity by directly neutralizing pathogens before they are able to infect of epithelial cells or invade tissues.^6^ In the airways, IgA antibodies dominate the response in the upper airways with increasing proportions or IgG toward the lung.^188^ Studies in which purified IgG and secretory IgA antibodies have been transferred into naive recipients to test their role in protection has been performed in influenza mouse models, and while their functions are of course not separate in the normal situation, these studies have concluded that while IgG is most important for protection of the lower airways, secretion of IgA is the dominating humoral mechanism protecting the upper airways.^189^ This difference is also reflected in the localization of plasma cells—in the upper airways IgA production dominate but in the lower parts both IgA and IgG producing cells are present.^190,191^ A later study suggested that both IgG and IgA in the lower airways have equal neutralizing capacity, suggesting a safeguard mechanism to protect against infection.^192^

Despite their role in protecting the airways from contracting contagious diseases, many questions regarding how B cell system function in the airways are unanswered. Among these are how cells from different inductive sites collaborate and which cell movements are needed for efficient responses. Furthermore, similar to the gut, the airways maintain a commensal microbiota during healthy conditions; NALT tissues are in direct contact with a rather rich commensal microbiota in the nasal and mouth cavities, whereas fewer bacteria are present in the lower airways and probably even less in the medLN that drain them.^193,194^ A forthright interpretation of the fact that IgA plasma cells dominate in the upper airways and IgG in the lower airways, and that there are separate inductive sites for the upper (NALT) and lower (iBALT and medLN) airways would be that the immune responses of two parts are strictly separate. However, why would then so many IgG expressing cells be present in NALT/tonsils when they are not present in the mucosa or glands of the upper airways^77,195,196,197^? And, since it takes more than one week after infection for iBALT to be generated—do they play any role in a for viral clearance during primary response^198^? It appears that GC B cells in iBALT have different antigen specificity and B cell repertoire as compared to GC B cells in medLN after viral infection.^199^ This could be due to distinct T cell signals between mucosal and lymphoid tissues, different local antigen availability or to other, yet unidentified, stimuli. The same study also identifies that a large majority of GC B cells in iBALT are non-Influenza specific, even when mice are infected with influenza. This could be attributed to the characteristic of iBALT as a general priming site, where cells of many specificities can be activated.^200^ Future studies will need to address this and investigate whether already formed GC in iBALT are permissive for activated B cell entry, such as we have described for GC in PP.^145^

Another potential, but more restricted, role could be for the generation of tissue-resident memory cells.^201^ After the discovery that there are non-circulating effector memory-like CD8 T cells that do not leave the lungs, similar CD4 and, more recently, B cells were also identified.^202–204^ Recently, a newly identified subset of Tissue-resident helper (Trh) cells, which are independent of Tfh in lymphoid organs, has been shown to be crucial for the generation of iBALT, antigen-specific B cells, and local antibody production.^205,206^ Further, these cells are particularly important for B cell differentiation to plasmablasts upon reinfection.

To better understand de novo response in the lower airways to an actual infection, we recently conducted a single-cell RNASeq study using a mouse influenza model.^207^ At 7, 14, and 28 days after intranasal infection of mice with Influenza type A virus, hemagglutinin(HA)-specific B cells from lung (including iBALT), medLN, and spleen were isolated, and we performed single-cell RNASeq analysis paired with single-cell B cell receptor cloning on these cells. This relatively unbiased setup allowed us to determine how a typical response to influenza developed over space and time in the lower airways, and how B cells belonging to antibody clones mutated and distributed over the tissues. Although all cell subtypes were present in all organs, the distribution of cells was different between organs with GC B cells dominating in spleen and medLN and memory B cells within lung. Interestingly, there were no drastic differenced in the composition of cells within an organ between day 7 and 28. Memory cells from lung differed from those in other organs by expressing *Cd69* and other genes suggestive for being tissue-resident memory cells. As judged by clonal relationships GC B cells, memory B cells, and plasmablasts within, but not between, organs shared origin. There was one notable exception from this—memory B cells in lung showed clonal relationships with GC and memory cells from both medLN and spleen. There was no apparent shift in the output from GC from memory B cells to plasmablasts; both cell types appeared to be generated all through the response. Neither did we see any the difference in affinity between the cells. However, plasmablasts more often belonged to large clones, whereas memory B cells showed a larger breath of antibody variants. Taken together, the data suggest that whereas GC responses are local in nature, those in iBALT are not sufficient to provide the lung with memory B cells and plasmablasts, but these also arrive from medLN and spleen. Thus, it appears that mucosal tissues in the airways, including lung, are a main site for memory B cell and plasmablast residency. These are maintained to protect the host from reinfection with the same or a variant pathogen.

## 10 Human Mucosal Responses to SARS-CoV-2 Infection

It appears that in individuals infected with SARS-CoV-2, the major site for viral replication early during disease are the upper airways.^208^ Despite this, most studies of SARS-CoV-2 immune responses have focused on IgG production and systemic responses. In fact, a recent review argued that the role of the mucosal system in the response to SARS-CoV-2 was a neglected area that should be further addressed.^209^ While this is still true, some data have been published that look more into the roles that airway responses and IgA plays. Some reports found that IgA antibodies reactive to SARS-CoV-2 are often detectable before both IgM and IgG in serumou,^11,12,210^ and it was later reported that IgA and IgM levels in serum appear to decay faster than IgG although other studies did not find this.^211–213^ It is still unclear if this represents a more rapid decline in production or not; an alternative scenario is that short-lived plasmablasts expressing either class are gradually dying at a similar pace, but that the titers of IgG will show a slower decay due to differences in serum half-lives of antibody classes.^20^ It is also important to note that the early antibody decay in serum of any class will not give relevant information about long-term production.^214^ In addition, serum studies will not be able to determine whether IgA antibodies are secreted locally. Some studies have described the secreted IgA antibodies in saliva as well as in BAL and breast milk,^210,215,216,217,218,219^ and although the levels of antibodies in these body fluids appeared to decline as rapidly as in serum, further studies are needed to determine the result long term as some plasma cells at mucosal surfaces can be very long-lived.^33,185^ Interestingly, high background titers of IgA binding to SARS-CoV-2 antigens were noted in some study, which could potentially be linked to cross-reactive antibodies from previous infections with endemic coronaviruses.^217–219^ When it comes to virus neutralization it is still unclear what role IgA plays. Some studies have reported that SARS-CoV-2-specific IgA antibody levels in serum show a better correlation with the ability of the serum to neutralize SARS-CoV-2 than IgG levels do,^210,220,221^ at least early during the response but other studies have come to the opposite conclusion.^155,222,223^ Another found that IgA in nasal washes efficiently neutralized SARS-CoV-2.^219^ Regardless, one study demonstrated that the ability of secreted IgA to form dimers increased its ability to neutralize SARS-CoV-2 more than 10-fold, making secreted mucosal antibodies highly efficient.^224^

Although data are now emerging regarding IgA production and its potential role during SARS-CoV-2 infection, less has been done to characterize to which extent antigen-specific cells are part of a mucosal responses or not. As the major site of infection is the upper airways, it is important to determine to which extent they are primed in tonsils and if they will home to these areas to protect. Several studies have noted a strong plasmablast response, and some have also described that many plasmablasts express IgA with the majority of these being IgA1 expressing.^210,225^ In one of these, higher expression of CCR10, a chemokine receptor associated with mucosal homing, was found on plasmablast compared with naive and memory B cells, with IgA^+^ plasmablasts also expressing higher levels than IgG^+^ ones.^210^ Another recently published study showed ongoing development of more mutated memory B cells after recovery and demonstrated that some gut epithelial cells may still be infected at this time, which lead them to suggest that gut antigens may then drive the response.^212^

**Figure 4:**
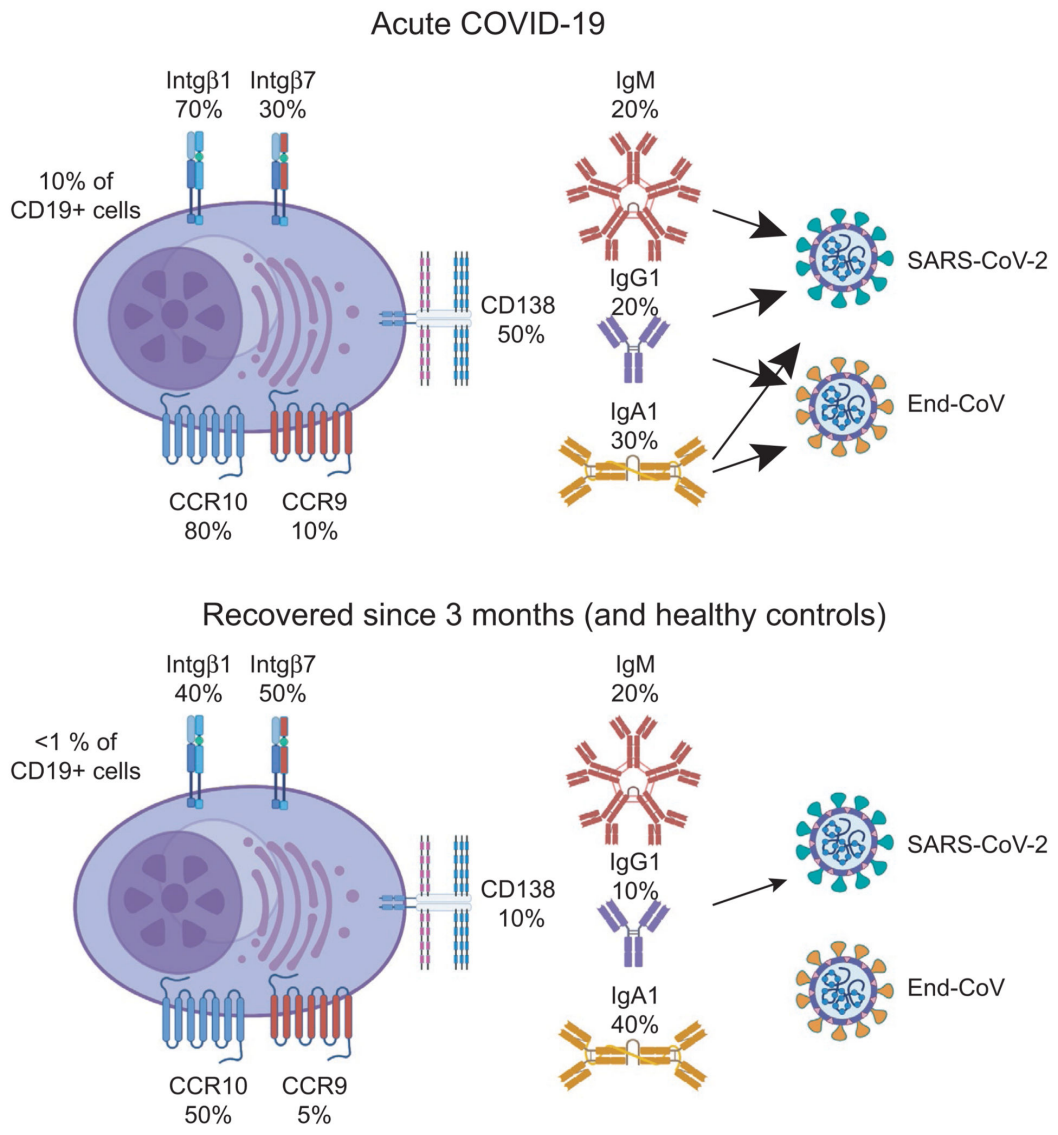
Plasmablast responses during COVID-19 disease. Plasmablasts responses were studied in hospitalized COVID-19 patients during acute disease and three months after recovery. Plasmablast made up 10% of all CD19+ B cells in blood during active disease, but less than 1% 3 months after recovery or in healthy controls. Plasmablasts encountered in blood during active disease expressed higher levels of integrin β1, CCR10, and CD138 than those after recovery, and produced antibodies of IgA1, IgG1, and IgM class, which suggested that they many generated in a mucosal immune response. Antibodies of all classes produced by the plasmablasts during disease reacted with SARS-CoV-2, and both IgA and IgG antibodies showed cross-reactivity to endemic coronaviruses. After recovery, the plasmablasts in blood produced low levels of IgG antibodies against SARS-CoV-2 while antibodies of other classes could not be detected, and they did not show cross-reactivity

We will very soon submit a study that address some of the issues discussed above (Figure 4) (Lundgren et al, manuscript in preparation). In this, we studied the plasmablasts response in COVID-19 patients during disease and after recovery and compared it to healthy adults. Using a set of monoclonal antibodies detecting all human antibody classes,^226^ we found that IgA1, IgG1, and IgM plasmablasts dominated the early response, and that three months after recovery the number of plasmablasts in blood had normalized. As many as 70% of all plasmablasts expressed integrin β1, with approximately 30% of these also expressing integrin β1, and 80% CCR10. CCR9 expression was relatively rare. Interestingly, an increased number of plasmablasts expressed CD138 during disease compared to steady state, a marker potentially linked to GC origin and/or longevity.^227,228^ Taken together, it appears that a large proportion of plasmablasts during SARS-CoV-2 disease suggestive of a mucosal response with airway homing a likely outcome. In addition to these observations, we also detected increased levels of memory B cells expressing CD45RB and CD69, a phenotype that suggests that they may develop into resident memory B cells.^229^

We also observed that antibodies produced by these early plasmablasts—both IgA and IgG—showed cross-reactivity to endemic coronaviruses. It has been suggested in previous studies that memory B cells generated during encounters with endemic coronavirus responses may be part of the response to SARS-CoV-2^230,231^ a situation that would be somewhat similar to the “original antigenic sin” observed during influenza.^232,233^ Interestingly, while cross-reactivity was readily detected during acute infection, neither the dwindling plasmablasts response against SARS-CoV-2 three months after infection or reactivity in memory B cells activated to antibody secretion showed cross-reactivity larger than background. Thus, whereas pre-existing cross-reactive memory may be recruited into the response early on, the response develops into higher specificity and less cross-reactivity over time, and the SARS-CoV-2 did not appear to replenish the memory against previously encountered coronaviruses to any large extent.

## 11 Conclusions

The development of the vertebrate immune system was influenced by the requirement to protect the semipermeable mucosal membranes that enables exchange of gases and nutrients.^234^ Here, in addition to protection from pathogens, the immune system needed to maintain a balance with commensal microbiota, which lead to the that specific secreted mucosal antibody classes developed already in bony fish.^4,5^ The duality that exists in the function of the mucosal immune system—maintenance of commensal strains and eradication of pathogenic ones—has fashioned its development all since. Here, we describe recent studies that show the mucosal system can respond to model antigens or novel pathogens, resulting in T cell-dependent IgA responses. We also discuss that the findings in these and previous studies indicate that most, if not all, IgA responses in adult individuals are generated through these pathways or at least are channeled into them. Thus, while truly T cell-independent IgA responses are certainly possible, they are unlikely to contribute much to overall IgA production in healthy adult individuals. Nevertheless, T cell-independent pathways may contribute during early life when the commensal microbiota is established.^235^ If future efforts in understanding IgA induction, production, and responses, it is important for researcher to be more careful in differentiating these scenarios.^236^ Germ-free mice reconstituted with a microbiota or immunodeficient mice into which purified cells are transferred are likely to be affected by homeostatic effects and/or redundancy and will be poor proxies of the situation in normal adult individuals already reacting to a complex microbiota. For researchers studying mucosal immunology to eventually reach a consensus view on how the IgA system protects against pathogens but at the same time maintains the commensal microbiota, it is important to perform studies in systems that resembles physiological conditions.

## Figures and Tables

**Table 1:** Arguments for and against that T cell-independent IgA responses play a major role in adult invidiuals

Argument for T cell-independent response	Argument for that it may not be relevant in healthy adults
LPS and polysaccharides, classical T independent antigens, trigger IgA responses at mucosal surfaces.	These are not encountered as single antigens at the mucosa but in combination with or even linked to proteins that could provide T cell help. When antibodies were cloned that were reactive against LPS in humans or polysaccharides in mice, these had gone through the process that introduce mutations in V regions—which is normally considered a tell-tale sign of having proliferated in GC.
B1 B cells transferred into immunocompromised hosts will start producing IgA in the gut.	The behavior of lymphocytes in hosts lacking competing cells is often unpredictable. When B1 and normal lymphocytes were transferred simultaneously, B1 cells were able to provide natural IgM in circulation but did not contribute to gut production.
Mice and humans that lack T cells or signals crucial for B-T interactions will still produce IgA in the gut, sometimes at close to normal levels, despite lacking GC that are typically associated with T dependent responses.	This may not be the case when competing with other pathways. When antibodies derived from gut IgA-producing plasma cells were cloned from such mice or memory cells from CD40L deficient humans, their V regions largely lacked mutations while essentially all V regions were mutated in normal individuals.
Studies have reported that IgA CSR may happen within the lamina propria outside of any organized lymphoid tissues.	Some of these studies were performed before ILF have been described and included these structures in the analysis. AID expression and cellular proliferation are closely linked to class- switch recombination, and several studies in mice and humans where the authors have carefully studied expression of AID, expression of proliferation markers, or molecular markers indicative of ongoing CSR to IgA have failed to detect any signs of it when ILF are carefully removed from the analysis

## Data Availability

Data sharing not applicable—no new data generated.
